# Integration of large-scale community-developed causal loop diagrams: a Natural Language Processing approach to merging factors based on semantic similarity

**DOI:** 10.1186/s12889-025-22142-3

**Published:** 2025-03-08

**Authors:** Melissa Valdivia Cabrera, Michael Johnstone, Joshua Hayward, Kristy A. Bolton, Douglas Creighton

**Affiliations:** 1 Institute for Intelligent Systems Research and Innovation, Deakin University, Waurn Ponds, Geelong, VIC Australia; 2 Global Centre for Preventive Health and Nutrition, Institute for Health Transformation, Deakin University, Geelong, VIC Australia; 3 Institute for Physical Activity and Nutrition, Deakin University, Geelong, VIC Australia

**Keywords:** Natural Language Processing, Semantic similarity, Systems thinking, Community, Health

## Abstract

**Background:**

Complex public health problems have been addressed in communities through systems thinking and participatory methods like Group Model Building (GMB) and Causal Loop Diagrams (CLDs) albeit with some challenges. This study aimed to explore the feasibility of Natural Language Processing (NLP) in simplifying and enhancing CLD merging processes, avoiding manual merging of factors, utilizing different semantic textual similarity models.

**Methods:**

The factors of thirteen CLDs from different communities in Victoria, Australia regarding the health and wellbeing of children and young people were merged using NLP with the following process: (1) extracting and preprocessing of unique factor names; (2) assessing factor similarity using various language models; (3) determining optimal merging threshold maximising the F1-score; (4) merging the factors of the 13 CLDs based on the selected threshold.

**Results:**

Overall sentence-transformer models performed better compared to word2vec, average word embeddings and Jaccard similarity. Of 161,182 comparisons, 1,123 with a score above 0.7 given by sentence-transformer models were analysed by the subject matter experts. Paraphrase-multilingual-mpnet-base-v2 had the highest F1-score of 0.68 and was used to merge the factors with a threshold of 0.75. From 592 factors, 344 were merged into 66 groups.

**Conclusions:**

Utilizing language models facilitates identification of similar factors and has potential to aid researchers in constructing CLDs whilst reducing the time required to manually merge them. While models accurately merge synonymous or closely related factors, manual intervention may be required for specific cases.

**Supplementary Information:**

The online version contains supplementary material available at 10.1186/s12889-025-22142-3.

## Background

In a world of complex problems and intricate societal challenges, we seek for methods to help us understand them and identify key leverage points for intervention and change. Complex problems are difficult to understand because the behaviours of the components interacting as a system cannot be predicted or reduced to their individual properties [1]. Some significant examples are global poverty, climate change, crime, obesity, and mental health. In mental health for example, one of the characteristics that makes it a complex problem, is the presence of social, environmental, and biological elements that can influence the mental illness of an individual. Other characteristics include the relationships between these elements that produce feedback structures and emergent behaviours making the drivers of ill mental health difficult to understand [2]. Understanding the complex nature of these problems requires a systemic approach that considers the multifaceted interactions and interdependencies between different components of the system [3]. 

Causal Loop Diagrams (CLDs) are graphical representations of the direct and indirect causal relationships between factors in a system, created to explicitly map the understanding of a problem. Causal relationships are represented as arrows directing cause, including its polarity, from one factor to another [4, 5]. 

Group Model Building (GMB) is a common participatory method that involves collaborating with groups of stakeholders (for example policymakers, grassroots community members and other partners) to build CLDs to understand, manage and address complex problems in their community or organisation [6]. This practice involves the development of CLDs which may be used, for example, to develop implementation strategies for policy recommendations, or evidence-informed actions and strategies to tackle the problem under consideration [7, 8]. 

GMB has been applied to studies of many different problems, such as, the perception of mental health among Syrian refugees in two Lebanese communities [9], healthy eating, active living and childhood obesity in 49 communities in the United Sates [10], the perceptions of 16–18-year-olds of the drivers of adolescent obesity in five European countries [11], understanding food behaviours, transportation and health in 10 Latin American countries [12], among others. In the above-mentioned studies, multiple GMBs with multiple participant groups were conducted. Participants from each group created their own CLD of the system, the models were then compared across the different groups and merged into one overarching CLD.

The main motivations for researchers to merge causal maps are to have a simplified model, which helps them analyse the problem more broadly, and highlight the commonalities and/or differences of the diverse points of view people have regarding the problem [13]. 

In a research context, for example, analysing perspectives of different communities serves to identify similarities between them and deepen the understanding of how a problem behaves in general. This way, one can find solutions or identify better places to act [14]. Another purpose for merging maps is to use the shared map as advocacy when trying to communicate a problem to stakeholders, government and/or funders. It may be more effective to present an aggregated high-level map of several communities rather than the views of just one. Aggregated high level results are more difficult for stakeholders to dismiss, as they outline the view of a group rather than individual experiences [15]. 

Even though CLDs have been successfully used to analyse complex systems, there are some challenges that need to be addressed due to the intricate structure of these diagrams. As per the analysis of Asif et al. [16], one of the most significant problems is to merge individual CLDs into one collective diagram. Simply putting together all the factors and loops from every individual map can end up in a very complex CLD, which may make it less obvious for stakeholders to visualise the problem. They argue that a simplified CLD that still captures the different points of view from the individual diagrams, is more useful for a better understanding of the system. As more factors are added to the model, the complexity increases, creating for example, more causal paths between factors and more structural clusters of factors in the network [5]. 

Previous studies have identified different methodologies to merge CLDs created by individuals or group of individuals into a collective CLD. This was achieved in the CO-CREATE project, where GMB was used in five European countries to create CLDs representing the perceptions of 16–18-year-olds of the drivers of adolescent obesity. The process in CO-CREATE was long and laborious with limitations such as relying on human judgement which could produce inconsistencies [11]. 

As the number of factors and maps increase, one key challenge in merging CLDs is the significant effort required to code all individual factors and the involvement of different people in the decision-making process. A promising method to reduce the level of manual effort and the objective of this study is to investigate the feasibility of using Natural Language Processing (NLP) to merge factors from causal loop diagrams created by different groups that have used the GMB process.

There has been a lot of recent development in NLP focused on model architecture and model pre-training to perform a wide variety of tasks. In the machine learning community, several open-source libraries to access large-scale pretrained models can be found. Some of the tasks performed by these models are sentence similarity, text generation and classification, sentiment analysis, question-answering, translation, among others [17]. 

Semantic textual similarity (STS) is a method that measures how similar in meaning two texts are, capturing deeper conceptual relationships between texts. STS can be applied in various fields, for example in qualitative research, text classification and clustering are used to analyse interview transcripts or surveys and identify thematic codes for categorising responses, thereby increasing efficiency and consistency. Another application is sentiment analysis, which helps group similar feedback, helping businesses improve customer satisfaction and decision-making [18]. To evaluate similarity, STS models convert texts into vectors called embeddings (a list of numerical values that encode information about the text’s meaning, derived from patterns learned in large collections of text) and calculate how close (similar) they are to each other using mathematical methods. A common approach is measuring the cosine angle between the two vectors, evaluating how closely the vectors align (the Euclidean distance, dot product and other trigonometric measures could also be used) [19]. 

Previous models like convolutional neural networks and recurrent neural networks used techniques like Word2vec, WordNet, support vector machine, and bag-of-word models, to understand background information from text, but they do not consider the surrounding context of the words [20]. Measures like Jaccard similarity and dice coefficient are considered as lexical similarity measures. These measures calculate the number of words that overlap between two texts, ignoring meaning and semantic context of words. According to Mudigonda et al. [21] the measures that consider semantics to calculate similarity are very close to human knowledge.

Freund and Giabbanelli [22] proposed an algorithm to combine nodes from individual causal maps based on semantic and structural information. To compute semantic similarity, they used Ontology Matching and a utility matrix to align words based on similarity score. The Ontology Matching technique uses Jaccard Similarity, which is a traditional algorithm based on literal matching of words. To determine the Jaccard Similarity, the authors utilized the WordNet lexicographical database to evaluate the semantic similarity between pairs of words within each factor of the causal map. Their proposed aggregation method successfully combined 22 individual maps into a coherent conceptual model. However, the research involved some manual intervention to address issues with certain factors not being recognized by the WordNet database. Hosseinichimeh et al. [23] introduced a computer program that applies a large language model to automate the generation of CLDs from textual data. They encountered the problem of having different variable names for the same concept, but they successfully applied cosine similarity to calculate the similarity between them.

Pretrained language models such as ELMo, GPT, BERT and XLNet have been applied to semantic similarity tasks with outstanding results, their attention mechanism allows them to prioritize specific words depending on the context [24]. Until today, BERT has achieved the highest performance in understanding syntax and semantics of short sentences, including the variant models created by fine-tuning or modifying the base model such as, AlBERT, RoBERTa, Sentence-BERT and DeBERTa [25]. 

There exists little research using algorithms from NLP to merge individual causal maps into a comprehensive one. This study will review the ability of applying pretrained language models to improve and reduce the manual efforts in the aggregation process of CLDs created by different communities through participatory workshops.

## Methods

Thirteen CLDs from different communities were created through GMB workshops as part of the VicHealth Local Government Partnership (VLGP), implemented in the state of Victoria, Australia. The detailed methodology is published elsewhere [26, 27]. Briefly, in each of the 13 communities, local council teams delivered 1–3 participatory GMB workshops, face to face or online, to groups of community stakeholders. These participants included children and young people, community leaders and members from local government, non-government organisations, commercial, education and health sectors. The CLDs from each council described the factors and the interrelationships influencing the health and wellbeing of children and/or young people in the community.

The maps were built with the systems mapping software program called Systems Thinking in Community Knowledge Exchange (STICKE) and the factors were downloaded using the same software for their subsequent analysis [28]. 

The following method was implemented for the analysis of the factors and merging of the CLDs with NLP models:

The first step was to identify duplicate values across all CLDs, including exact matches of factors that appeared in different maps. The second step was to extract a list of unique factor names to subsequently preprocess them for further testing. This process involved spelling checks, removal of punctuation, numbers and symbols that did not affect the meaning of the factor, ensuring consistency in capitalisation and replacing abbreviations with their complete forms. These steps were implemented to emphasize relevant results that are similar but not identical, while also lowering the computational burden of the subsequent NLP steps.

In the third step, different pretrained language models were tested to determine the similarity between pairs of factors. The algorithm creates embeddings, which are numerical representations of each factor, and uses them to calculate a similarity score between the two factors, ranging from 0 (no similarity) to 1 (identical meaning).

Models like BERT and RoBERTa have achieved state-of-the-art performance on tasks like STS. They however require a very high computational overhead, as the embeddings they produce are for each word (or token), not sentences. Sentence-BERT (SBERT) is a modified version of the pretrained BERT network that derives semantically meaningful sentence embeddings much faster, taking seconds instead of hours [29]. The creators of SBERT developed the ‘sentence-transformers’ library with different models that have a very high performance on STS tasks. Different sentence-transformers models were used to perform the initial calculation of the similarity between the factors. The comparisons with the highest similarity scores were analysed by one of the subject matter experts (SME), who was the lead researcher implementing systems thinking in the VLGP project. The SME established the ground truth, marking each comparison as either 1 (if the factors should be merged) or 0 (if they should not be merged).

The fourth step was to apply an optimiser to calculate the optimal threshold (x) that maximises the F1-score for this dataset. The threshold determines the cutoff for deciding whether a similarity score indicates a match (merge) or not. Based on the ground truth, the similarity score and the optimal threshold, the optimiser categorises each data point as: true positive (TP): correctly identified as a merge; false positive (FP): incorrectly identified as a merge; true negative (TN): correctly identified as not a merge; or false negative (FN): incorrectly identified as not a merge (see Table 1). The F1-score is a performance metric that calculates the harmonic mean of precision (the proportion of correctly identified merges out of all predicted merges) and recall (the proportion of correctly identified merges out of all actual merges). It evaluates how well the model balances incorrect merges (false positives) and missed merges (false negatives) [30].

**Table 1:** F1-score matrix

Ground truth	Score > x	Score < x
1	TP	FN
0	FP	TN

Ultimately, the model that achieved the highest F1-score was used to merge the factors from the 13 CLDs into groups based on the given threshold.

## Results

The 13 CLDs initially had a total of 670 factors, however, after the duplicate values were identified, 592 unique factors remained. As the focus of the study was to merge similar factors from different communities / source maps, the comparisons from the same source maps were deleted, leaving 161,182 comparisons for the analysis.

As an initial analysis, sentence-transformer models were compared against other similarity models like Word2vec, average word embeddings and Jaccard similarity.

The models in Table 2 produce high quality sentence embeddings and their performance is better compared to other models that have not been optimised for the tasks of semantic search or cross-domain sentence encoding. The average performance is evaluated on how well the models can turn the sentence into an embedding that captures the meaning of the sentence and how well the models can understand this meaning and match them to other sentences based on semantics [31]. 

The average word embeddings use the embedding method depicted in Table 2 under the ‘Base Model’ column to represent words as vectors based on similar meanings or contexts. They were assessed as comparison because according to Reimers [31] their computation speed is much higher than the transformer-based models but the quality of the embeddings are worse. The all-* models were evaluated as they were trained on all available data of more than 1 billion pairs. They furthermore perform better than other traditional methods like Word2vec, on encoding sentences and searching semantics on diverse tasks from different domains.

**Table 2:** Pre-trained models evaluated on semantic search and sentence encoding

Model Name	Base Model	Training Data	Average Performance
all-mpnet-base-v2	mpnet-base	all available training data (over 1 billion training pairs)	63.3
all-distilroberta-v1	distilroberta-base	all available training data (over 1 billion training pairs)	59.84
all-MiniLM-L12-v1	MiniLM-L6-H384-uncased	all available training data (over 1 billion training pairs)	59.76
multi-qa-distilbert-cos-v1	distilbert-base	215 million question-answer pairs	59.41
paraphrase-multilingual-mpnet-base-v2	paraphrase-mpnet-base-v2	trained on parallel data for 50 + languages	53.75
average_word_embeddings_glove.6B.300d	Word Embeddings: GloVe	-	-
average_word_embeddings_levy_dependency	Word Embeddings: Levy et al.	-	-

After the initial assessment of the similarity models, a disadvantage found of Word2vec and average word embeddings is that they do not give a score or give a value of zero to some factors. This occurs when none of the words are present in the Word2vec vocabulary, or the training data does not contain significant co-occurrence patterns or contextual relationships between the words. Although the acronym “LGBTQIA+” and the abbreviation “COVID19” are well known today, they are not well represented by these models. In comparison to the transformer-based models that are trained on billions of data and can comprehend these types of words.

Table 3 compares the score given to some factors, where a poor score is given by models that do not consider semantic similarity and a high score by models that do (transformer-based). As the Jaccard similarity algorithm is based on literal matching of words, also gives some inconsistent results, not considering the actual meaning of the factor.

**Table 3:** Examples of similarity scores given to some factors by different models

Factor 1	Factor 2	Ground truth	Sentence-transformers	Word2vec	Average word embeddings	Jaccard similarity
lgbtqi+	lgbtqia+	1	0.95	0	0	0
isolated socially	social isolation	1	0.87	0.54	0.49	0
e-cigarettes	vaping and e-cigarettes	1	0.82	0	0	0.33
covid	covid19	1	0.79	0	0	0

The next step was to compare the different transformer-based models against the ground truth from the SME. As 161,182 comparisons were increasingly complex to analyse, only the comparisons with a score above 0.7 from the sentence-transformers models paraphrase-multilingual-mpnet-base-v2, all-mpnet-base-v2, all-MiniLM-L12-v2, multi-qa-distilbert-cos-v1, all-distilroberta-v1 were considered for the manual analysis by the SME.

After a thorough analysis of whether two factors should be merged or not merged, an optimisation method was used to maximise the F1-score by optimising the threshold. Table 4 summarises the optimal threshold and the corresponding F1-score for each of these models.

**Table 4:** Optimal threshold and F1-score for different similarity models

Model	x	F1-score
paraphrase-multilingual-mpnet-base-v2	0.75	0.68
all-MiniLM-L12-v2	0.67	0.67
all-mpnet-base-v2	0.62	0.66
multi-qa-distilbert-cos-v1	0.60	0.65
all-distilroberta-v1	0.51	0.63
average_word_embeddings_levy_dependency	0.38	0.61
average_word_embeddings_glove.6B.300d	0.06	0.60
Jaccard similarity	0.14	0.58

The model with the best F1-score, paraphrase-multilingual-mpnet-base-v2, was used to merge the factors from the 13 CLDs according to the threshold of 0.75. From a total of 592 factors, 344 were merged into 66 groups, while the remaining 248 factors were left unmerged as unique entries. Table 5 exemplifies some of the factors that were merged into one group due to their similarity in meaning. In two instances, we identified inconsistent factors merged into one group, as one general factor linked several that were similar in meaning. Group 5 in Table 5 exemplifies this case, where the factor “affordability” was merged with e.g., “affordability of health food” and “housing affordability”, merging all the related factors into one big group.

**Table 5:**
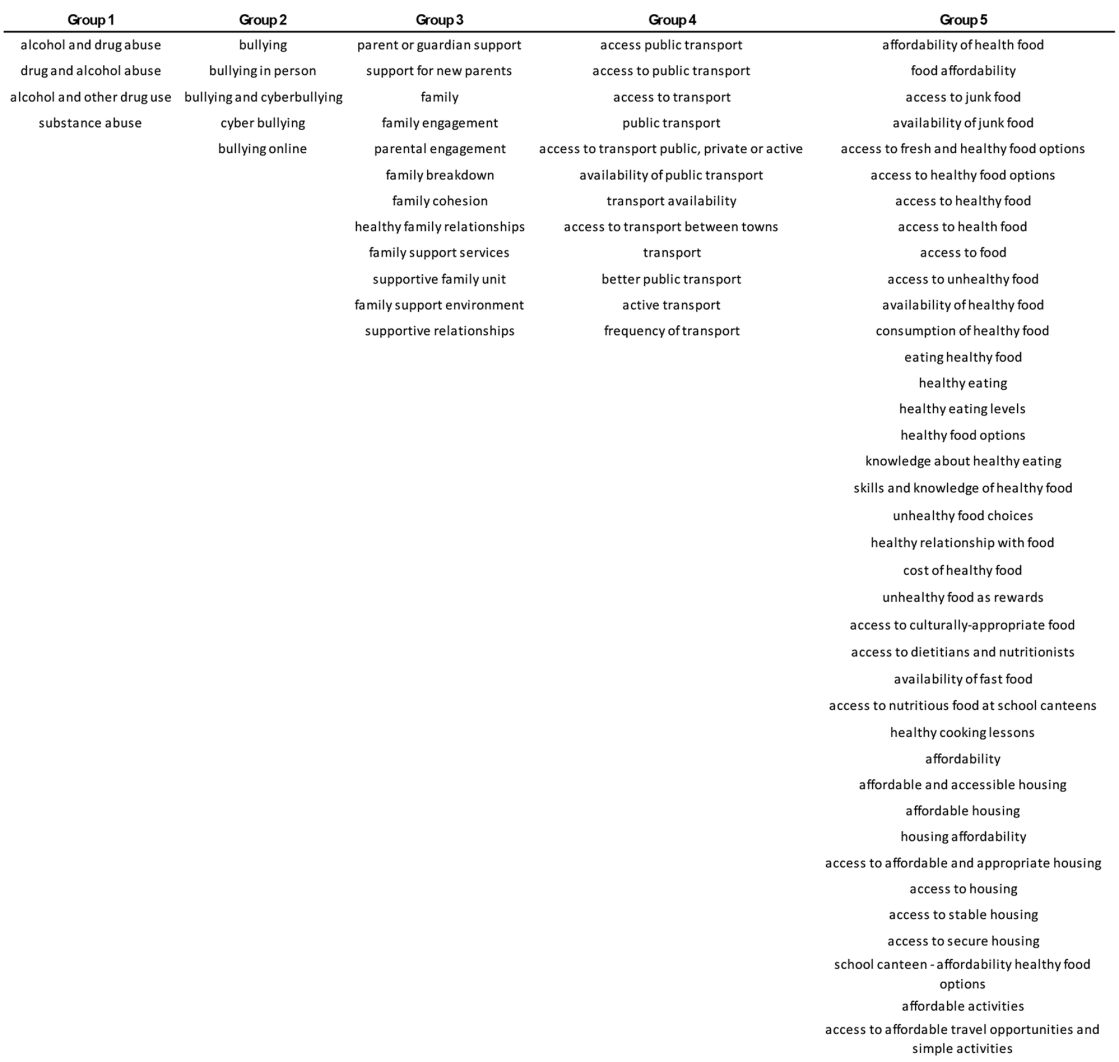
Examples of factors merged into groups based on their similarity

## Discussion

The results demonstrate that NLP, specifically the use of sentence similarity models, is a promising area to guide researchers on possible factor merges in a CLD. In terms of model accuracy, even though Jaccard similarity and average embeddings have good results when the words are the same in a sentence (even if they are in different order), the transformers models still outperform them. Transformers can capture the similarity in wording as well as in meaning making them more suitable for this task.

On the other hand, there are important issues encountered through this study that should be addressed in future work to optimise this method. Similarly, Amur et al. [20] identified some challenges that short-text semantic similarity (STSS) has in comparison to longer sentences. They are more difficult to understand, and it is more difficult to identify the semantics based on their context. The drawbacks of STSS can be summarised as follow: ambiguity/limited amount of information (up to 5 words); polysemy and sparsity (terms or phrases with several meanings, sophisticated and vague concepts); anomalies (abbreviations, non-standard terms, misspellings, grammar and punctuation errors, and slang); sentence structure (run-on sentences and sentence fragments). Some of these challenges, along with specific examples from our analysis, are explained below.

As an initial step, it was concluded that preprocessing the factors was extremely important as there were several abbreviations, spelling mistakes and no value-added data. The models are pre-trained for certain general abbreviations, such as, “e.g.” (for example) and symbols like “&” (and), but the data used in this study had very specific abbreviations such as “MCH” (maternal and child health) and “YP” (youth programs), where it was important to change them to their complete form, for the models to perform better. To identify exact matches of factor names, this step was also important, as one community misspelled the word “stimatisation” without the “g” and another did not.

Giabbanelli et al. [32] discusses the challenge of merging individual maps into one due to the variations in language across the participants. They argued that limiting the participants to choose from a pre-established list of concepts prevents the emergence of terms that were not previously considered. This is why in GMB it is important to allow participants to choose their own factors, but it is fundamental that moderators guide them to define them correctly.

According to Kim [33] there are guidelines and suggestions on the mechanics of creating causal loop diagrams, including the selection of factor names. He states that nouns must be chosen instead of verbs and action phrases. For example, “Costs” is better than “Increasing costs” because the polarity of the links already indicates whether it decreases or increases relative to the other factor. In our data, we encountered similar issues, where the factors were not defined correctly, and they lacked neutrality. For example, “poor role modelling” should have been defined as “role modelling” only, as the polarity of the connections already indicates if they have a positive or negative impact on the other factor.

Another problem we encountered with the data was the limited amount of information given when defining the factors. Projects like the VLGP allow communities to be empowered to do their own GMBs, giving researchers a lot of insight into the communities’ perspectives at scale. But, while reducing the reliance on “experts” is good from a community development perspective, it does have an impact on data quality. For example, the factor “accessibility” does not specify what the accessibility relates to. When compared to “digital accessibility” the merge could be right or wrong depending on the context that was given in the workshops. Another example is the comparison between “power of family” and “supportive family unit”, they probably have a link, but the first factor was poorly defined, and it was difficult to interpret what the participants meant exactly by “power”.

Another important point to note is that in some instances, factors with opposite meaning are merged by the SME. As mentioned before, a neutral name is preferred and depending on the factor it is connected to, the polarity of the links can be changed. For example, if “food security” and “food insecurity” are merged into “food security”, the polarities connected to the factor “food insecurity” would have to be reversed. In terms of the accuracy of the models, in cases where the wording was similar, such as the factors mentioned before, they give them a high similarity score. In other cases, such as “culturally appropriate environments” vs. “language and cultural barriers”, the SME considered them as the same construct with opposite framing, while the model gave a similarity score below the threshold.

Sometimes we also encountered both problems, when a factor was poorly defined and when it could possibly be merged to its opposite. For example, if “LGBTQIA+” is assumed to mean inclusivity, it could be merged with “homophobia”, but the SME decided not to as it was not well named, and the model gave them also a low score.

Another instance where the model might struggle to correctly merge the factors is when they focus on similar words or topics and not specifically on the structure of the predicate in a sentence. For example, “access to health services” emphasizes the action or ability to obtain health services, while “knowledge of how to access health services” focuses on understanding/knowing the process of accessing health services. The availability of a service does not always mean usage if there is not knowledge/awareness of how to access it. Another example is when small details are important to define a factor according to the community’s story of the CLD. From a general perspective “access to health services” is similar to “access to free healthcare”, but here it was important to differentiate the “free” aspect from the private healthcare.

In a CLD, specific details that define causality are important, for example “availability of healthy food” leads to “consumption of healthy food”. For this reason, the SME decided to keep them as separate factors, while the model considered them very similar. On the other hand, when we compare “consumption of healthy food” to “eating healthy food” both the SME and the models consider them similar as their synonymity is straightforward.

When a factor is very broadly defined, we might also encounter some issues. “Access to food” is very general, when compared to other factors, it would make sense to merge it with “access to healthy food” and “access to culturally appropriate food”, but if you analyse these last two, they do not exactly mean the same and therefore shouldn’t be merged.

When we encountered inconsistencies in factors merged into one group due to a general name, such as “affordability” (see Table 5), it could be beneficial to look at the factors they are linked to. This could potentially give more context to the story the participants were aiming for this factor.

The approach of merging maps after running GMB sessions is frequently based on the expertise and knowledge gathered from these sessions by the researchers conducting the study. Reviewers with content expertise analyse the meaning of each factor to identify and remove the ones that are redundant or merge similar factors into a new one with a more generalised name. As this approach is based on human judgement, researchers involved in the study agree that it may not produce accurate and/or consistent results. It is also very time consuming, analysing each factor, coding, and merging it, especially when dealing with hundreds of them. However, by leveraging NLP techniques, researchers can simplify and accelerate this process. As a result, this approach facilitates the creation of a simpler diagram that communities can use to arrive at actionable insights more quickly.

### Lessons learnt for future directions

The use of language models can help greatly in the automation process and identification of similar names when dealing with hundreds of factors from several CLDs. As seen in Table 5, there are several examples where the model accurately merged factors that mean the same and factors that are very similar in meaning. The similarity models have potential to help researchers identify different factors that could be collapsed into one, simplifying the causal diagrams significantly. As mentioned before, there are also some specific situations where the models do not perform correctly and still do not gather some details that a SME would by doing it manually. When grouping the factors in terms of similarity, it could, however, suggest to the SME or participants which factors to merge, making the process less manual and leaving the final decision to the user.

An important lesson from this study is the potential limitation of relying on a single SME to establish the ground truth for merging variables across maps. Ideally, this process would involve multiple members of the core modelling team who were actively involved in the workshops where the maps were originally created. Such a collaborative approach would ensure that diverse perspectives and a shared understanding inform the merging decisions.

In this study, the reliance on a single expert was a practical necessity, as only one individual had comprehensive insight across all the maps. Now that the feasibility of the NLP process has been established, a critical next step would be to evaluate the reliability of the human-based merging process, to better assess the potential for NLP-based map merging in future applications.

In terms of quality issues, they are to some degree for now, a necessary outcome of increasing communities’ access to GMB as a tool. Projects intending to use NLP for merging maps could ensure that factors are clearly and consistently defined during GMB sessions to improve NLP compatibility. Future research could explore the use of language models to guide communities in refining their choice of language when building CLDs, potentially enhancing alignment with automated merging methods and be integrated into platforms like STICKE.

Potentially, with further advancements in NLP, we could also find more accurate models for short text similarity or train them once more data becomes available.

## Conclusion

The use of NLP techniques accelerates the merging of CLDs aiding communities in constructing simplified, generalizable models of complex health problems. Given the rapid evolution of NLP, ongoing research in this area is essential to continue supporting communities in constructing CLDs and advancing health prevention efforts globally.

## Electronic supplementary material

Below is the link to the electronic supplementary material.

Supplementary Material 1

## Data Availability

The datasets used and/or analysed during the current study are available from the corresponding author on reasonable request.

## Abbreviations

CLD: Causal Loop Diagrams
GMB: Group Model Building
NLP: Natural Language Processing
STS: Semantic Textual Similarity
DEMATEL: Decision-Making Trial and Evaluation Laboratory
VLGP: VicHealth Local Government Partnership
STICKE: Systems Thinking in Community Knowledge Exchange
SME: Subject Matter Experts
TP: True Positive
FP: False Positive
TN: True Negative
FN: False Negative
SBERT: Sentence-BERT
STSS: Short-Text Semantic Similarity
