# Supplementary material for: Integration of large-scale community-developed causal loop diagrams: a Natural Language Processing approach to merging factors based on semantic similarity
Source: BMC Public Health. 2025 Mar 8;25:923. doi: 10.1186/s12889-025-22142-3 (PMC11889750; doi:10.1186/s12889-025-22142-3)
Supplement: Supplementary file 1 — Supplementary Material 1 [file 12889_2025_22142_MOESM1_ESM.pdf]

Set ID	Factor name	Set ID	Factor name
1	alcohol and drug abuse	17	gaming
1	drug and alcohol abuse	17	online gaming
1	alcohol and other drug use	17	playing with people online
1	substance abuse	18	access to education
2	bullying	18	access to school
2	bullying in person	18	easier access to education
2	bullying and cyberbullying	18	access to educational facilities or option
2	cyber bullying	18	access to community education
2	bullying online	18	education
3	parent or guardian support	18	engagement in education
3	support for new parents	18	positive engagement in education
3	family	18	school involvement
3	family engagement	18	attending school
3	parental engagement	18	going to school
3	family breakdown	18	education retention
3	family cohesion	18	value of education
3	healthy family relationships	18	value of education in the community
3	family support services	18	attendance at school
3	supportive family unit	18	school attendance
3	family support and environment	19	mentors
3	supportive relationships	19	positive mentors
4	active transport	19	mentorship of young people
4	transport	20	early intervention
4	access public transport	20	early intervention services
4	access to public transport	21	socio economic level
4	access to transport	21	socioeconomic status
4	public transport	21	socio-economic disadvantage
4	access to transport public, private or active	22	invasion of privacy
4	availability of public transport	22	privacy
4	transport availability	23	social media use
4	access to transport between towns	23	use of social media
4	better public transport	23	social media
4	frequency of transport	23	social media addiction
5	affordability of health food	23	social media pressures
5	food affordability	23	targeted social media campaigns
5	access to junk food	24	e-cigarettes
5	availability of junk food	24	vaping and e-cigarettes
5	access to fresh and healthy food options	24	smoking or vaping
5	access to healthy food options	25	community violence
5	access to healthy food	25	violence
5	access to health food	25	family violence
5	access to food	25	reported family violence

5	access to unhealthy food	25	awareness family violence
5	availability of healthy food	25	domestic violence
5	consumption of healthy food	25	youth violence
5	eating healthy food	25	witness violence between adults
5	healthy eating	26	poor role modelling
5	healthy eating levels	26	poor role models
5	healthy food options	26	good role modelling
5	knowledge about healthy eating	26	role modelling
5	skills and knowledge of healthy food	26	role models
5	unhealthy food choices	26	healthy role modelling
5	healthy relationship with food	26	role modelling of parents or carers
5	cost of healthy food	27	confidence
5	unhealthy food as rewards	27	self confidence
5	affordability	27	level of self-esteem
5	access to culturally-appropriate food	27	self worth and self respect
5	access to dietitians and nutritionists	28	safety
5	affordable and accessible housing	28	safety in public
5	affordable housing	28	community safety
5	housing affordability	29	cycle of trauma
5	access to affordable and appropriate housing	29	trauma
5	access to housing	30	accessibility
5	access to stable housing	30	digital accessibility
5	access to secure housing	31	language barriers
5	access to nutritious food at school canteens	31	language and cultural barriers
5	school canteen - affordability healthy food options	32	family expectations
5	affordable activities	32	family pressure or family expectations
5	healthy cooking lessons	33	addressing the issue of homelessness for young people
5	access to affordable travel opportunities and simple activities	33	homelessness
5	availability of fast food	34	local professionals
6	literacy and reading levels	34	number of local professionals
6	literacy levels	35	gender discrimination
6	literacy	35	gender equity
7	community activities and events	35	gender diversity
7	community events	35	discrimination
7	knowledge of community activities	35	gender stereotypes
7	community connection	35	stereotype
7	community connection and support	35	gender roles
7	access to community support	36	access to sugary drinks
7	social connection	36	sugary drink consumption
7	social connection levels	37	body image
7	social interaction	37	positive body image
7	social engagement	38	diversity of education opportunities

7	peer and social interactions	38	education about diversity
7	relationships with peers	39	identity
7	appropriate opportunities for social connection	39	sense of identity
7	social opportunities	39	sense of self
7	socialising with peers	40	food education
7	appropriate social activities	40	food literacy
7	socially active	41	celebrations and visibility of cultural awareness
7	community groups	41	cultural awareness
7	access to exercise	41	cultural diversity
7	access to exercise facilities or gyms	41	embracing diversity
7	exercise	41	cultural backgrounds
7	physical activity	41	culture
7	exercise options	41	cultural education and celebration
7	physical activity levels	41	cultural inclusion
7	fitness	42	community based resilience
7	unstructured physical activity or sport	42	resilience
7	non sporting activities	43	household income
7	access to sport	43	income
7	access to sport and recreational activities	44	advertising
7	access to organised sport	44	promotions and advertising
7	participation in sport	45	lgbtqi+
7	participation in sport or organised activity	45	lgbtqia+
7	participation in local sports and activities	45	lgbtqia+ inclusion
7	participation in non organised sport or activities	45	lgbtqia+ encouragement
7	sport	45	positive mental health of lgbtqia+ youth programs
7	team sports	46	work
7	sport options	46	work skills
7	limited opportunities for a range of sports	46	skills
7	fun activities and social sport options	47	life experiences
7	informal sport	47	shared lived experiences
7	being physically active in the community	48	excess screen time
7	peer support	48	screen time
7	access to health services	48	excessive tech use and screen time
7	access to healthcare	49	amount of homework
7	knowledge of how to access health services	49	doing homework
7	access to free healthcare	50	access to early childhood education
7	mental health	50	supported transition to early years education
7	mental ill health	51	apprenticeship opportunities
7	mental health problems	51	work or apprenticeships

7	poor mental health	51	employment
7	mental health well	51	workforce
7	mental health wellness	51	employment opportunities
7	mental health and wellbeing	51	jobs available
7	mental wellbeing	51	local employment opportunities
7	access to mental health services	51	employment - shift work
7	access to mental health support	51	future career opportunities
7	availability and access to mental health support	52	financial problems
7	mental health services	52	financial stress
7	wellbeing	52	anxiety
7	mental health unwell	52	stress and anxiety
7	access to mental health assessment	52	stress
7	mental health workshops	52	study-related stress
7	physical wellbeing	53	sexual assault
7	knowledge of positive mental health practices	53	violence against children and sexual assault
7	physical health	54	activities after school
7	mental health in schools	54	lack of activities to do after school
7	engagement with health services	54	out of school hours activities
7	health literacy	55	service reliance
7	mental health literacy	55	services capacity
7	access to opportunities	56	leadership development
7	variety of activities	56	young person’s leadership opportunities
7	doing physical activity at home	57	access to places and programs for art and creativity
8	negative stigma	57	access to the arts
8	stigma	58	climate change
9	adequate sleep	58	global warming
9	quality sleep	59	age or year level
9	sleep	59	your age or under 18
9	sleeping	60	food insecurity
9	sleeping patterns	60	food security
10	access to services	61	peer pressure
10	access to services and programs	61	social pressure
10	access to support services	62	access to parks
10	knowledge of support services	62	access to public green spaces
11	youth voice	63	volunteer opportunities
11	youth voice representation	63	volunteering opportunities
11	empowering youth voice	63	volunteering opportunities and work experience
12	opportunities for youth participation	63	volunteering hub
12	youth participation	64	first nations heritage
13	parent education	64	traditional owners and first nations
13	parental education attainment	65	access to safe youth spaces
13	parent involvement in children's education	65	youth drop-in spaces

14	disordered eating	66	infrastructure e.g. roads footpaths street lighting
14	eating disorders	66	streetlights
15	covid		
15	covid19		
15	covid-19 impacts		
15	covid cases		
16	isolated socially		
16	social isolation		
16	social exclusion		
16	isolation		
16	disconnection or isolation		
16	geographic isolation		
